# CPR Training for Nurses: How often Is It Necessary?

**Published:** 2012-02-01

**Authors:** J Mokhtari Nori, M Saghafinia, M H Kalantar Motamedi, S M Khademol Hosseini

**Affiliations:** 1 Faculty of Nursing, Baqiyatallah University of Medical Sciences, Tehran, Iran; 2 Department of Anesthesia, Trauma Research Center, Baqiyatallah University of Medical Sciences, Tehran, Iran; 3 Trauma Research Center, Baqiyatallah University of Medical Sciences, Tehran, Iran

**Keywords:** Training, Cardiopulmonary resuscitation (CPR), Knowledge, Skill, Nurse

## Abstract

**Background:**

The ability to respond quickly and effectively to a cardiac arrest situation rests on nurses being competent, prepared and up-to-date in the emergency life-saving procedure of cardiopulmonary resuscitation (CPR). This study aimed to determine the extent to which nurses acquire and retain CPR cognitive knowledge and psychomotor skills following CPR training courses.

**Methods:**

A quasi-experiment was used. CPR knowledge of 112 nurses was assessed via a questionnaire using valid multiple-choice questions. An observatory standard checklist was used and CPR performance on manikins was evaluated to assess psychomotor skills (before the course baseline, after the course, after 10 weeks and then 2 years after the 4 hours CPR training course). Scores were based on a scale of 1 to 20.

**Results:**

A mean baseline score of 10.67 (SD=3.06), a mean score of 17.81 (SD=1.41) after the course, 15.26 (SD=3.17) 10 weeks after and 12.86 (SD=2.25), 2 years after the 4 hours CPR training course was noticed. Acquisition of knowledge and psychomotor skills of the nurses following a four-hour training program was significant. However, significant deterioration in both CPR knowledge and psychomotor skills was observed 2 years after the training program among 42 nurses.

**Conclusion:**

The study findings present strong evidence to support the critical role of repetitive periodic CPR training courses to ensure that nurses were competent, up to date and confident responders in the event of a cardiac arrest.

## Introduction

Cardiopulmonary resuscitation (CPR) is a critical component of basic life support (BLS) and the established first-line of response to a cardiac arrest in the interim before defibrillation and advanced life support (ALS) are performed.[1] CPR has the potential to save lives in life threatening emergencies such as stroke, respiratory arrest, trauma, drowning and airway obstruction.[1] CPR is associated with survival and can prevent impending death.[1] Thus, nurses’ competency in CPR is a critical factor in patient outcome from cardiac arrest. CPR competency is defined as possessing cognitive knowledge and psychomotor skills to be able to perform CPR in a cardiac arrest situation.[2][3] This study further defines CPR competency as encompassing both the acquisition and retention of CPR cognitive knowledge and psychomotor skills. Despite the fact that nurses’ ability to perform CPR may be a critical determinant of patient survival from a cardiac arrest, there is compelling evidences to suggest that registered nurses across continents lack competence in the performance of a proper CPR.[2][4][5] The literature is clear that CPR skill cannot be acquired or retained easily by nurses.[5][6][7][8][9] Studies report different levels of competency in individual CPR skill components and that they vary dramatically along a range from 0% to 100%.[4][8] Core CPR skills (performing proper chest compressions) ranked lowest in psychomotor skills.[4][8]

While there are a number of factors associated with CPR competency, one critical factor is CPR instruction.[10] The aim of CPR training is to ensure that nurses not only acquire CPR knowledge and skills, but that they also retain this knowledge to be able to respond competently and confidently to a lifethreatening cardiac arrest situation.[2] In other studies, it has been shown that CPR knowledge and psychomotor skills are difficult to retain.[1][9]

Experiences showed that in many critical situations, staff personnel (medical doctors or registered nurses etc) do not have sufficient basic CPR knowledge with time.[10] This study aimed to determine the extent to which nurses acquire and retain CPR cognitive knowledge and psychomotor skills following CPR training courses in several time frames.

## Materials and Methods

This quasi-experimental study was done from January 2006 to January 2008. The degree of the knowledge and psychomotor skills of 112 nurses were assessed in 4 stages (before training, immediately after training, 10 weeks and 2 years after training). In stage four, 42 participants were enrolled.

Lectures and theoretical trainings regarding cardiac arrest and basic CPR, reasons of cardiopulmonary arrest and modes of prevention and basic CPR were discussed for 2 hours. Then, 100 minutes of practical exercises were done on the manikins. For assessment, a questionnaire with 20 multiple-choice questions was designed, and was validated by 10 members of an expert panel. The highest knowledge score possible was 20. A valid observant checklist of CPR skill assessment according to IHF (Irish Heart Association in 2000) was used for assessment of psychomotor skills. The highest possible score was 26. Received information from 4 stages (before training, after training, 10 weeks and 2 years after training) were compared with each other and were analyzed by SPSS software (version 16, Chicago, IL, USA). For comparing average scores, the repeated measure of ANOVA test was used.

## Results

The mean before training was 10.95, immediately after training was 17.96, and10 weeks after training was 16.12 and 2 years after training was 12.86. Significant difference between three stages was observed with p<0.001. However, in the fourth stage, the test was undertaken by 42 nurses as 70 nurses were transferred to other hospitals.

The mean scores of psychomotor skills before training was 4.86, immediately after training was 24.41, and 10 weeks after training was 21,46 and 2 years after training was 9.26 while there was a significant difference between the stages with p<0.001 However, significant deterioration in both CPR knowledge and psychomotor skills was observed 2 years after the training program in 42 nurses (70 nurses aborted the study) (Table 1)

**Table 1:** The mean and standard deviation of knowledge test scores and psychomotor skills scores before training, immediately after training and re-test (10 weeks and 2years after training).

**Kind of test**	**No**	**%**	**Mean**	**SD**	**Test* P* value**
Knowledge pre-test	112	54.75	10.67	3.06	Repeated measures ANOVA **
Knowledge post-test	112	89.80	17.81	1.41	
10 weeks after Knowledge post-test	112	80.60	15.26	3.17	**
Knowledge after 2 years	42	64.30	12.86	2.25	*P*<0.001
Psychomotor skills pre-test	112	18.70	5.38	5.24	
Psychomotor skills post-test	112	93.30	24.55	1.3	
10 weeks after psychomotor skills post-test	112	82.50	21.5	4.38	
Psychomotor skills after 2 years	42	36.81	9.57	5.46	

## Discussion

CPR skill retention is defined as retaining the knowledge capacity to perform CPR effectively at a certain point in time after CPR training.[8] The retention of CPR knowledge and skills is a key factor in determining CPR competence.[2] However, there are universal evidences to suggest that CPR knowledge and skills are poorly retained across populations.[2][9][10] In a seminal review of studies on CPR retention over a 9-year span (1980–1989), poor CPR retention skills were noticed.[10]

Findings related to the degree of knowledge and psychomotor skills in 4 stages in our study showed that the degree of CPR knowledge and psychomotor skills before training indicate that, although all nurses received CPR training during their study in the university, most of them lacked CPR cognitive knowledge in the pre-test.

In this research, 54.75% of subjects responded correctly to the knowledge questions. Our findings indicated that nurse’ psychomotor skills in CPR before training were low as the average psychomotor skills scores in pre-test was 4.86 (about 18.7%). In the study of Nagashima and colleagues, CPR practical ability of nurses was 17%.[11] Broomfield and Devlin showed that even the nurses who had work authorization, they did not have sufficient competence in skills of CPR.[3]

Greig and colleagues in their study reported that most of the personnels had weak competence in CPR skill and differences of competence levels ranged from 0% to 100% and the CPR skill scores in “performing cardiac compression” was low.[9] Other researchers also indicated the weak ability of nurses in CPR.[3] The degree of CPR skill and knowledge immediately after training indicated that the degree of knowledge immediately after re-test increased considerably. In several studies, the increase of nurses’ knowledge after retaining was emphasized too.[1][6][12][13][14][15][16]

Psychomotor skills scores immediately after training increased from 18.7 to 93.9. Many researchers reported the effect of re-test on CPR practical ability.2,3,14 CPR knowledge was retained more than CPR practical psychomotor skills.

cSeveral authors showed that CPR skill decreased with time. They demonstrated that retention ability of CPR skill in training was weak.[2][5][9][13][14][15][16][17][18][19] The extent to which nurses’ CPR knowledge and psychomotor skills were retained 2 years after re-test decreased from 80.6% to 64.3%. Timsit and colleagues found that one year after re-test, the knowledge of those who had re-test was more than those who did not.[19]

Because of the weak retention of CPR knowledge, researchers emphasized on the necessity of periodical training.[5][15][9][17][18][19] After 2 years, the degree of psychomotor skills decreased from 82.5% in 10 weeks after training to 36.81%. Decrease in psychomotor skills was 29.39% more than knowledge skill. The necessity of regular CPR training periods for retention of knowledge and psychomotor skills were the important findings of our study. This study like others supported CPR training to increase the competency and to ensure an effective CPR in cardiopulmonary emergency events.
